# The Bible and Materia Medica

**Published:** 1855-02

**Authors:** 


### THE BIBLE AND MATERIA MEDICA.

When the last hour comes to me, when in that upper chamber, long past midnight, the flickering light burns lonelily, and passing forms noiselessly and quick too plainly show that death is there, when the bleak winter's wind whistles from without, or sends its melancholy moan through the lattice, alternating with the groan of the dying; when the softest tread and the slightest whisper fall harshly on the last sense;* when feeling and sight, and taste, and speech, all are gone, but immortal thought, the more immortal as it shakes away its mortal shackles, still lives in the freshness of eternal youth, in such an hour, when this present body shall have been wasted to a skeleton, this hand palsied of its strength, this eye glazed with the film of the grave, this cheek blanched with the last chill, this forehead, high and white, and broad and clear now, shall be thickly studded with the dew-drops of death, and this tongue falters out the last farewell to the dear ones around, so long loved and labored, and cared for, when such an hour comes to me, I want to feel the ineffable consolation, that something said or something done, some line written, some sentence published, some page composed, some sentiment recorded shall live after me, which shall in its influences continue to benefit and bless some candidate for the skies, to the last hour of recorded time. Feeling thus, now and heretofore, I desire to repeat of THE BIBLE that:

A nation would be truly happy, if it were governed by no other laws than those of this blessed book:

It is so complete a system that nothing can be added to it or taken from it:

It contains everything needful to be known or done:

It affords a copy for a king, and a rule for a subject:

It gives instruction and counsel to a senate, authority and direction to a magistrate:

It cautions a witness, requires an impartial verdict of a jury, and furnishes the judge with his sentence.

It sets the husband as lord of the household, and the wife as mistress of the table—tells him how to rule, and her how to manage.

* It is said that the hearing is the last sense to die.

It entails honor to parents, and enjoins obedience to children.

It prescribes and limits the sway of the sovereign, the rule of the ruler, and the authority of the master; commands the subjects to honor, and the servants to obey; and promises the blessing and protection of the Almighty to all that walk by its rules.

It gives direction for weddings and for burials.

It promises food and raiment, and limits the use of both.

It points out a faithful and eternal guardian to the departing husband and father—tells him with whom to leave his fatherless children, and in whom his widow is to trust—and promises a father to the former, and a husband to the latter.

It teaches a man how to set his house in order, and how to make his will; it appoints a dowry for his wife, and entails the right of the first born, and shows how the younger branches shall be left.

It defends the right of all, and reveals vengeance to every defaulter, over-reacher and oppressor.

It is the first book—the best book, and the oldest book in the world.

It contains the choicest matter—gives the best instruction; affords the greatest pleasure and satisfaction that ever was enjoyed.

It contains the best laws, and most profound mysteries that ever were penned; it brings the best of tidings, and affords the best of comforts to the inquiring and disconsolate.

It exhibits life and immortality from everlasting, and shows the way to glory.

It is a brief recital of all that is past, and a certain prediction of all that is to come.

It settles all matters in debate, resolves all doubts, and eases the mind and conscience of all their scruples.

It reveals the only living and true God, and shows the way to him, and sets aside all other gods, and describes the vanity of them, and all that trust in such; in short, it is a book of laws, to show right and wrong; a book of wisdom, that condemns all folly and makes the foolish wise; a book of truth, that detects all lies and confutes all errors, and a book of life. that shows the way from everlasting death.

It is the most compendious book in the world—the most authentic, and the most entertaining history that ever was published.

It contains the most ancient antiquities, strange events, wonderful occurrences, heroic deeds, unparalleled wars.

It describes the celestial, terrestrial, and infernal worlds, and the origin of the angelic myriads, human tribes, and devilish legions.

It will instruct the accomplished mechanic, and the most profound artist.

It teaches the best rhetorician, and exercises every power of the most skillful arithmetician; puzzles the wisest anatomists, and exercises the wisest critic.

It corrects the vain philosopher, and confutes the wise astronomer; it exposes the subtle sophist, and makes diviners mad.

It is a complete code of laws, a perfect body of divinity, an unequalled narrative—a book of lives—a book of travels, and a book of voyages.

It is the best covenant that ever was agreed on—the best deed that ever was sealed—the best evidence that ever was produced—the best will that ever was made, and the best testament that ever was signed. To understand it, is to be wise indeed; to be ignorant of it, is to be destitute of wisdom.

It is the king's best copy, the magistrate's best rule, the house-wife's best guide, the servant's best directory, and the young man's best companion; it is the schoolboy's spelling-book, and the learned man's masterpiece.

It contains a choice grammar for a novice, and a profound mystery for a sage.

It is the ignorant man's dictionary, and the wise man's directory.

It affords knowledge of witty inventions for the humorous, and dark sayings for the grave, and is its own interpreter.

It encourages the wise, the warrior, the swift, the over-comer; and promises an eternal reward to the excellent, the conqueror, the winner, and the prevalent. And that which crowns all, is that the author is without partiality, and without hypocrisy.

"In whom is no variableness or shadow of turning."

Who composed the above description of the Bible, we may never know. It was found in Westminster Abbey, nameless and dateless; no doubt its author now is a blest inhabitant of heaven, as all will be who love it, as he seems to have done.

I have thus drawn the attention of my readers to a Book which some of them may have neglected, as not being up to the age in which we live. This is a great mistake. Human nature and human need are the same now as in Adam's day, and will continue the same, till time shall be no more. The principles of the Bible are exceeding broad, and cover the universe of men and things, reaching to all conditions of mortal life; if these principles were understood, and loved, and practised, there would be no need of a "*Journal of Health*" like mine, because those principles practised from youth, would forestall disease. The Bible reasons of "*Temperance*" as the means of avoiding "*judgment to come;*" declaring that "*there is no law against such*" as practise it; and that coming next in importance to "*knowledge*," it prepares the intelligent for the highest enjoyment of human happiness, being as it is, the foundation of human health.

With this "*temperance*," reaching to all things, we are enjoined to *exercise*, there being "*six days in which men ought to work*," and "study to work with (their) own hands," since "*if any would not work, neither should they eat*," and that instead of spending their time in discussing the business of other people and meddling with the concerns of their neighbors, they "*should work with quietness, and eat their own bread*," it no doubt being understood, that it was not their own, until it was earned.

Here then are the two fundamental rules of healthful life laid down with a precision and a directness which no intelligent mind can resist, that by personal labor, men should earn what they enjoy, and in that enjoyment, they should practise temperance with the guarantee of an exemption from "*judgment*" and "*law*," from suffering and punishment. Let every reader of the "*Journal*" then, aim for that happy, that blessed condition of mind, which receives every declaration of the Bible with the most implicit, the most unhesitating confidence, as meaning just what it says, having no disposition to equivocate or get around its plain injunctions by ingenious conjectures, or "*better renderings*." Doing so, you will be temperate and industrious, and conscientious, and as a matter of course, *healthy and happy;* then, you need be a subscriber to the Journal of Health no longer; you can then save that dollar, and with it, buy two bibles to give to some brother mortal, too poor to purchase one for himself, and dying, you will, with one hand resting on that book, the other pointing heavenward, feel

> "This little Book I'd rather own,
> Than all the gold and gems
> That e'er in monarch's coffers shone,
> Or all their diadems.
>
> Yes! were the seas one chrysolite,
> The earth one golden ball;
> And diamonds too the stars of night,
> This Book were worth them all."

In the heading of this article, I coupled the Bible with "Materia Medica," that is, with a free translation, *all the medicinal articles in the world;* for as a means of health, it is worth them all, because the practical observance of its principles as to temperance, industry, and cleanliness, would secure physical health to nine-tenths of the human family, to the age of three score years and ten: while that calmness and equanimity of mind, which is the necessary result of an unwavering reliance on the promises of the Bible, would secure a deliberation and a presence of mind in times of suddenly threatened calamity, which would make casualties requiring surgical aid of very rare occurrence. The man or woman who is a christian from sterling principle, founded on a habitual reading and study of the Scriptures, is not alarmed from his propriety in the battle and the breeze, in the pestilence of midnight, and the crashing fury of a noonday tornado; he feels abidingly, *my Father is there,* "*of whom shall I be afraid;*" with such an abiding trust, he can calmly look around him at a moment when death's missiles fly thick as hailstones, and choose, if any, the best way of escape. He feels, if he escapes, it is well, if not, he is prepared to go to his Father.

Does novel reading have that effect? Does it give bravery to meet life's stern realities, to breast the storm in the hour of shipwreck, or dare the almost certain death of the chamber of festering pestilence, and the rankling plague? Oh, no! the poor creature, if strength is left, vies with the whirlwind's flight; or more probably, is petrified with fear, and stands aghast, immovable as a monument of stone, or falls to the earth with more than an infant's helplessness.

I am a physician, and am necessarily not unfamiliar with death. Amid whole barrels of lungs in London, I have dug out with eager fingers, in scientific delight, "*beautiful specimens*," in medical phrase, of the "perfect cicatrix," the "healed cavity," the "ghastly abscess," the "calcareous mass," the "solidified lobe," and all that; and in the schools of Paris, have seen the naked dead, all but the face; that, reader, is always covered in the dissecting-room; no long custom can make us familiar with that sad and shocking sight,—have seen the naked dead brought in upon the shoulders, or in a bag or barrel, and strewn around the floor, as carelessly as a child dashes down a toy, for a dollar a piece—yes, a dead man or woman, for a dollar; or here at home, have seen at the end of a lecture season, whole pyramids, as high as the ceiling, of legs and arms, and trunks, and heads from many different bodies, thrown in one indiscriminate heap; some of them soon to be thrown into a hole and covered up, the others to be "macerated" or boiled, and strung together as a skeleton for some after "young gentleman" to handle and to study over. But these things do not appal the physician; he looks at them scientifically, and therefore rather likes the sight; it is instructive, and he does not hesitate to handle them, and gaze at them as nearly as the shortest pug will admit; but all this applies to cases where death has done his work on the stranger victim. But change the scenes, go back an hour, let the victim still have the breath of life, and let the bystander be that victim's physician, and the difference is wider than daylight and darkness: responsibility is summoned, sympathy is appealed to, professional skill is invoked, and while the heart weeps for the dying fellow mortal, the intellect blushes for its own helplessness. But to come more nearly to the point, there is another difference in the dying chamber, and it is infinitely wide; the difference in dying with a Bible and without one. I have seen them both, many a time and oft, and have as often felt, *it is worth the effort of a lifetime, to be able to die well.* By dying well, I mean, having a firm reliance on the truth of the Bible, that reliance having been made up of the myriads of convictions which have occurred in a previous life of christian rectitude. To witness such a death is glorious. It is a heart-lesson for good, which a century can not erase. But on that other picture, the poor dying creature, who has no Bible, no hope, no God! who feels himself dying, and yet says, "Doctor, I won't die!" "Get a carriage for me, and I'll soon be well as ever," and then he talked to me of his plantation, of his plans for clearing more ground, and the estimated product of each additional acre. "Send for my factor," said he, "and tell him to bring my account current."

"But, my friend, you may die to-night."

"I tell you, Doctor, you don't understand my case."

I sent for the factor, and for the satisfaction of those at home, whom he was never to see again, I sent for the minister too, who like the good man that he was, came right away; but he said, "*it is too late*," offered a prayer and left us alone. Scarcely had he gone, when the factor came; the very sight of the long bill of sales and per contra, waked up the last slumbering energies of the godless one, and after an examination, detecting an error of a few cents in an account involving many thousands of dollars, handed it for rectification, turned over, and died!

But in the *progress* of disease, the Bible is the best emollient; it makes the timid lion-hearted, and nerves the wasted body with a strength almost superhuman. "I have preached," said the lamented Spencer, "and when I reached home, I found my boots part filled with blood," and yet so engaged was he in his Master's work, that none of all his loving and loved people, thought that he was ill. At a later hour some one said, "your pains must be agonizing?" "Agony! it is far short of what I feel." Yet, not a murmur, not a complaint ever escaped the good man's lips.

Many people say that *old age* requires a stimulant, that a little wine or brandy, "now and then," would be of great service, would brace up the system and supply a vigor, not to be attained in any other way. Whatever may be the advantages of stimulants to the aged, I know that my mother's mother took none, and yet beyond the age of three score and ten, she had the cheerfulness of a girl in her teens. I cannot recollect, that during all the years of our childhood, we ever paid her a visit, that we did not find the little stand at her right hand, and Scott's family Bible upon it, most always open, or spread upon her lap. She would knit awhile, then read a verse or two, with its explanation; and for every occurrence of life, whether of gladness, or of gloom, she had some pertinent scripture expression, that seemed to have a *dovetail* fit, as a cabinetmaker would say, I myself having been a kind of amateur in that line, at such odd times as it was found a bore, and that the mind would not work at orthography, etymology, syntax and prosody, for so early as then, I had observed when I was not in a studying mood, there was no use in trying. I never could do a thing when I did not feel like it. Reader, make a note of this, and it will do you and the world some good hereafter. *Be at it when the fit is on you;* then it is most likely to be done well, and what is worth doing at all, is worth doing well.

"*As I was saying*," Hannah Pyke was a cheerful old woman, and her love of the Bible, and of doing good, made her so. How many and many a dollar of hers went to "Princeton," a name as dear to her, as Jerusalem to a Jew. How many a bank note stuck to a minister's hand when she bade them good-bye; reader, they say "good-bye" "*out west;*" if you were to say *adieu* out there, they would think something was wrong in the upper story. How many a poor preacher went away from her hospitable home, with a coat or vest, or other garment that he did not bring with him, nor knew it was among his worldly goods, until with delighted surprise it was observed to be among the contents of the old "*saddlebags*" as they were spread out on the floor, husband and wife and little ones all around in a ring, each one hoping father had brought an apple, or cake of sugar, or a picture-book, or something else—how many of such presents were made, can only be known at the judgment, where both giver and receiver have long since gone. There were two other things my grandmother read occasionally; she took the "*Missionary Herald*" from the start, and that square, dumpy, queer kind of a newspaper, the "Boston Recorder," then the only religious newspaper in America. The Bible and her own experience told her what God was doing for her; these two papers told her what He was doing for the world outside, and what the young "studients," as she used to call them, were doing, whom she had placed in the ministry, in whole or in part; a few of whom, have had no equals; they too, now gone with the world's largest honors and most affectionate remembrances upon them. Now with these instances which, as it were, have grown up before my eyes, how can I do otherwise than to recommend to every young man and woman, who wishes to be healthful in mature life, and to pass on to a cheerful and painless old age—*the reading of the Bible as a means of health.* Some people I know, will turn up their noses with contemptuousness at such an idea, but such should remember, that sometimes contempt is mutual; and then again, their experiences are all on one side. I give one fact out of a million, and I know that the sentiment is true, from a wide observation; it is a moral demonstration, as conclusive and as clear, as any of Euclid's, and the sneers of a universe cannot bring shame to my face when I know that I am right. I might go further and give a "*recipe*" for reading the Bible with a view to its healthful influences, but for the charge of invading my minister's premises, to whose discourse on the first Sabbath in January, the world is indebted for any of good there may be in this tedious article. Still, I will lay aside a Doctor's dignity, and as "an humble friend of mankind" in general, and of the young in particular, I will make a suggestion as to the best way of reading the Bible with a view of a *daily gaining influence* over the principles and practices of our lives. Do not make an effort to read it all through in a year. Do not resolve you will read two or three chapters a day, nor one, necessarily; but do resolve, that you will read any number of verses from one to ten, *the first thing in the morning*, and think about what you have read all the time you are dressing; and get into a habit of fixing the mind on some one sentiment advanced, to be thought of several times during the day; a day will seldom pass, that observation will not confirm that sentiment, and thus every day will add an argument for Bible truth, until, before you are aware of it, your principles and your practices will be shaped by its dew-distilling teachings, and every word of it will be received with that childlike fearlessness, which none but an heir of immortality can ever know, and the possession of which confidence, is worth more than all worlds.

